# Ectopic pregnancy management and treatment strategies: A nationwide survey of Czech gynecological facilities

**DOI:** 10.1177/17455057261435815

**Published:** 2026-04-18

**Authors:** Borek Sehnal, Jan Zapletal, Michael J. Halaska, Krystof Brecka, Barbora Svedova, Martin Hruda, Anna Babkova, Lukas Rob, Petr Waldauf

**Affiliations:** 1 Department of Obstetrics and Gynaecology, University Hospital Kralovske Vinohrady and Third Faculty of Medicine, Charles University, Prague, Czech Republic; 2 Department of Anaesthesia and Intensive Care Medicine, University Hospital Kralovske Vinohrady and Third Faculty of Medicine, Charles University, Prague, Czech Republic

**Keywords:** ectopic pregnancy, methotrexate, tubal pregnancy, salpingotomy, interstitial pregnancy, cesarean scar pregnancy, cervical pregnancy, Czech Republic, nationwide survey

## Abstract

**Background:**

Ectopic pregnancy (EP) remains a significant cause of maternal morbidity and mortality, with diverse clinical presentations and substantial variability in treatment strategies.

**Objectives:**

To assess current EP management approaches across gynecological inpatient departments of all sizes in the Czech Republic.

**Design:**

A nationwide cross-sectional observational questionnaire study.

**Methods:**

A survey was conducted in January 2025. All 92 registered inpatient gynecological wards in the Czech Republic were invited to participate in an anonymous online questionnaire on EP diagnosis and treatment. A total of 89 departments (96.7%) responded. Facilities were grouped into four categories based on annual hysterectomy volume. Statistical analyses included chi-squared and Fisher’s exact tests with adjustments for multiple comparisons.

**Results:**

Methotrexate (MTX) was available in 82.8% of high-volume centers, compared with 48.3% of smaller facilities (*p* = 0.002). Despite this, 46.1% of institutions reported never using MTX. The use of MTX for all types of EP was significantly more common in larger centers (72.4% versus 45.0%, *p* = 0.023), although its use for tubal EP remained generally low (18.0%) with no significant difference by center size. Surgical treatment of tubal EP was consistently preferred by 36.0% of facilities, and 38.2% of surgeons occasionally performed salpingotomy. Laparoscopy was the dominant approach for interstitial pregnancies (86.5%), with universal adoption in high-volume centers (*p* = 0.007). Suction and curettage were most commonly used for cesarean scar (47.2%) and cervical (60.7%) pregnancies, although treatment strategies varied considerably. Observational management was preferred in pregnancy of unknown location with declining human chorionic gonadotropin (hCG) levels (83.2%), while diagnostic laparoscopy was favored when hCG levels plateaued or rose (57.3%).

**Conclusion:**

EP management, even within a country with a homogeneous healthcare system, may demonstrate considerable heterogeneity, strongly influenced by institutional size. These findings highlight the need for national guidelines to ensure standardized and evidence-based care.

## Plain language summary

How hospitals in the Czech Republic treat pregnancies outside of the womb: A nationwide survey of doctors’ approaches

Ectopic pregnancy is a serious medical condition in which a pregnancy develops outside of the womb, most often in the fallopian tube. If not treated properly, it can be life-threatening for the mother. This study examined how doctors in hospitals across the Czech Republic manage different types of ectopic pregnancies. Researchers sent a questionnaire to all 92 gynecology departments in the country and received responses from 89 of them. The goal was to understand how treatment decisions vary depending on the size and resources of each hospital. The study found that treatment approaches differ widely. Surgery is still the most common treatment for ectopic pregnancies, especially in smaller hospitals. A medicine called methotrexate, which can treat the condition without surgery, was available in most large hospitals but used in only 18% of cases overall. Some less common types of ectopic pregnancy, such as those located in a cesarean scar or cervix, were often referred to larger hospitals because smaller facilities lacked experience with these rare and complex cases. Overall, the study revealed a lack of consistency in how ectopic pregnancies are treated in the Czech Republic. While some hospitals have access to more advanced treatments, others rely mostly on surgery. The authors suggest that national guidelines should be established in every country to ensure that all women receive safe, effective, and consistent care—no matter where they are treated.

## Introduction

Ectopic pregnancy (EP) is defined as the implantation of a fertilized ovum outside of the uterine cavity, specifically beyond the endometrial lining. EP is a major contributor to maternal morbidity and accounts for an estimated 5%–10% of all pregnancy-related deaths globally.^1^ Clinical presentation typically occurs in the first trimester and is often characterized by nonspecific symptoms such as lower abdominal pain and vaginal bleeding. These symptoms may mimic other acute conditions—including appendicitis, urolithiasis, early pregnancy loss, or trauma—frequently resulting in diagnostic challenges.^2^ Notably, the prevalence of EP among women presenting to emergency departments has been reported to be as high as 18%.^1^

The literature identifies numerous risk factors associated with ectopic pregnancy, including maternal age over 35 years, smoking, prior pelvic or tubal surgery (including tubal ligation), a history of subfertility or infertility, the use of assisted reproductive technologies, the presence of an intrauterine device at conception, prior exposure to diethylstilbestrol, Black ethnicity, and pelvic inflammatory conditions, including rare parasitic infections affecting the genital tract.^2,3^

### Terminology definitions

Tubal ectopic pregnancy, defined as implantation of the fertilized ovum within the fallopian tube, remains the most common form of ectopic implantation, accounting for approximately 88%–96% of all ectopic pregnancy cases.^1,4–6^ Interstitial pregnancy is characterized by implantation of the gestational sac within the interstitial portion of the fallopian tube as it traverses the myometrium at the uterine fundus.^6^ This condition is frequently misnamed as cornual or angular pregnancy. The term cornual pregnancy should be reserved for intrauterine implantation within an anomalous uterine horn (cornu).^4,5,7^ Cesarean scar pregnancy (CSP) is defined as implantation on a previous cesarean section scar (type 1, endogenous) or within the scar/niche (type 2, exogenous). Cervical pregnancy (implantation within the endocervical canal), ovarian pregnancy (implantation within ovarian tissue), intramural (implantation within the myometrium), and abdominal pregnancy (implantation within the peritoneal cavity, most commonly in the omentum) represent rarer forms of EPs.^1,4–8^ Some authors have proposed new classification and diagnostic criteria for subserosal pregnancy, a rare variant of intramural pregnancy in which a portion of the gestational sac is covered only by the uterine serosa (Stabile2024-Subserosal). A special entity is heterotopic pregnancy, in which an intrauterine pregnancy (IUP) coexists with an extrauterine implantation.^1,4,7,8^

### Epidemiology

**Figure 1.**
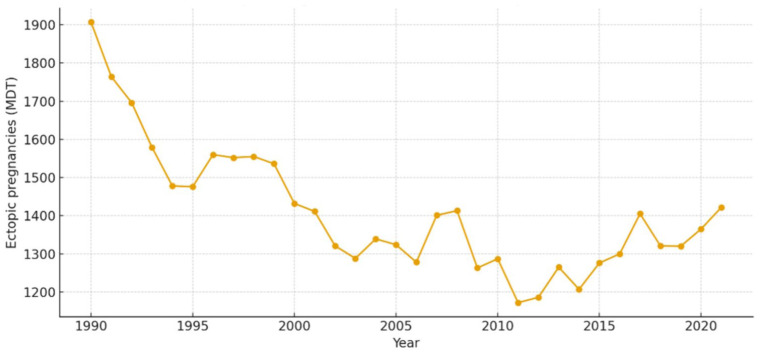
Trends in ectopic pregnancy in the Czech Republic (1990–2021).

According to the latest global estimates, the age-standardized incidence rate (ASIR) of EP from 1990 to 2019 was approximately 9.69 cases per 100,000 individuals worldwide.^9^ In Central Europe, the estimated ASIR in 2019 was 117.39 per 100,000 women (95% uncertainty interval (UI): 89.64–151.53).^9^ In the Czech Republic, mandatory reporting to the central health authority—the Institute of Health Information and Statistics of the Czech Republic (Ústav zdravotnických informací a statistiky (ÚZIS))—includes comprehensive national surveillance of all induced abortions, spontaneous miscarriages, and reported cases of ectopic pregnancy. In 1990, a total of 1907 ectopic pregnancies were documented. Since that time, a gradual decline has been observed, with the most recently processed data from 2021 indicating 1422 cases (Figure 1).^10^ After adjustment for the national age distribution, the ASIR for ectopic pregnancy was estimated at 68.2 per 100,000 women (95% UI: 64.7–71.6) in the Czech Republic, which has a population of approximately 11 million. The estimated annual percentage change (EAPC) in the ASIR of ectopic pregnancy is −0.89% (95% confidence interval (CI): −1.20% to −0.59%), reflecting a slightly steeper decline than the overall trend observed in Central Europe (EAPC −0.35%, 95% CI: −0.71 to 0.02). By contrast, increasing EAPCs for ectopic pregnancy have been reported in Western Europe (0.68%, 95% CI: 0.58 to 0.79) and Eastern Europe (1.44%, 95% CI: 0.85 to 2.04).^9^ These divergent patterns may have two explanations. First, the Czech Republic has maintained a long-standing system of compulsory reporting of all ectopic pregnancies, ensuring near-complete case ascertainment even more than 30 years ago. Second, following the political transition of the early 1990s and subsequent liberalization of societal norms, the use of hormonal contraception increased substantially. Consequently, the number of induced abortions performed on request up to 8 weeks of gestation has declined more than fivefold since 1990 (Supplemental Figure S1).^10^ This decline likely contributed to the gradual reduction in the incidence of ectopic pregnancy in the Czech Republic.

Historical data from an analysis of 1800 EP cases demonstrated 93% tubal localization, followed by 3.2% ovarian, 2.4% interstitial, and 1.3% in other anatomical locations.^11^ However, recent studies suggest a shift in the distribution of EP implantation sites. The incidence of CSP shows the most pronounced increase, paralleling rising rates of cesarean delivery and the expanding use of assisted reproductive technologies.^1,4,12–14^ A contemporary retrospective analysis of 1559 EP cases between 2003 and 2022 reported a decrease in tubal pregnancies to 88.5%, with a concurrent rise in non-tubal localizations: 5.6% interstitial, 3.8% cesarean scar pregnancies, 1.0% cervical, 0.9% ovarian, and 0.2% in other locations.^6^

### Diagnostic methods

Beyond serial human chorionic gonadotropin (hCG) measurements and transvaginal ultrasonography, several novel diagnostic approaches for ectopic pregnancy are being explored. Recent reviews highlight a range of experimental serum biomarkers—including activin-A/AB, pregnancy-associated plasma protein A, ADAM-12, vascular endothelial growth factor, and various microRNAs—some of which have shown promising sensitivity and specificity for distinguishing ectopic pregnancy from failing IUP in women with a pregnancy of unknown location (PUL).^1^ In addition, endometrial sampling with histology or frozen-section examination has been investigated as an adjunct tool to avoid unnecessary methotrexate (MTX) administration in misclassified intrauterine miscarriages.^1,15^ However, these strategies remain largely confined to research settings; most studies are small, heterogeneous, and lack external validation, and none of these markers has entered routine clinical practice. Currently, ectopic pregnancy is diagnosed almost exclusively on the basis of high-resolution transvaginal ultrasonography, which allows correct identification at the initial examination in approximately 75%–82% of cases.^8,16,17^ Repeated transvaginal ultrasonography combined with monitoring of serum hCG dynamics increases diagnostic sensitivity to 87%–99% and specificity to 94%–99%.^5,17^ To our knowledge, no centers in the Czech Republic routinely use additional serum biomarkers or endometrial sampling protocols for the diagnosis of ectopic pregnancy outside of individual research projects.

### Treatment options

Management of tubal and non-tubal ectopic pregnancies involves three principal strategies: observational management, surgical intervention, and pharmacologic treatment.^4,17^ These approaches may be employed independently or in combination, depending on clinical circumstances. Key factors influencing therapeutic decisions include the patient’s hemodynamic status, symptomatology, the location and size of the ectopic gestation, serum levels of hCG, and the presence of embryonic cardiac activity. Nevertheless, treatment practices may vary considerably even within the same geographic region and are frequently shaped by the clinical expertise and experience of healthcare providers. These, in turn, are often associated with institutional characteristics such as size, available resources, and case volume^1,4^

Expectant (observational) management may be employed in hemodynamically stable, asymptomatic women with tubal ectopic pregnancy who demonstrate a declining serum β-hCG level. Systemic (intramuscular) or local administration of MTX can be used not only for tubal ectopic pregnancy but also for virtually all other types of EPs. MTX therapy achieves a 75–90% success rate in appropriately selected patients, with higher efficacy reported for multidose regimens.^1,15,17–19^ A recent meta-analysis demonstrated comparable success rates between MTX (79.3%) and expectant management (68.6%; risk ratio (RR) 1.16; 95% CI 0.95–1.40) when the pregnancy is unruptured and serum hCG levels are below 2000 IU/L.^18^

Promising pharmacologic alternatives under investigation include the aromatase inhibitor letrozole, the Epidermal Growth Factor Receptor (EGFR) tyrosine-kinase inhibitor gefitinib, and the antiprogesterone mifepristone, particularly when combined with MTX.^20–22^ However, current evidence remains insufficient to recommend any medical therapy over expectant management for tubal ectopic pregnancy.^23^ Surgical treatment consists of either complete removal of the fallopian tube (salpingectomy) or a linear incision of the fallopian tube and excision of the ectopic gestation (salpingostomy).^15,17^ Whereas in developed countries virtually all ectopic pregnancy surgeries are performed laparoscopically, with few exceptions, an open surgical approach continues to predominate in developing regions. For example, an analysis of data from 17 countries in Africa and Latin America found that laparotomy was the most common surgical management for ectopic pregnancy (87.2%). In addition to limited technical resources, the main contributing factor was delayed diagnosis, as signs of peritoneal irritation due to hemoperitoneum were already present in half of the patients.^24^ In conclusion, among all available approaches, salpingectomy is statistically the most reliable treatment modality for tubal ectopic pregnancy.^23^

There is currently no global consensus regarding the optimal treatment of CSP.^4,6,13,17,25,26^ A systematic review from 2016 identified 14 different therapeutic strategies, with five being recommended: (1) vaginal resection, (2) laparoscopic excision, (3) hysteroscopic resection, (4) suction and curettage (S&C) combined with uterine artery embolization, and (5) hysteroscopy with embolization.^12^ While simple S&C is relatively reliable, it carries a hemorrhage risk of up to 28%. This risk can be significantly reduced to 4% when combined with uterine artery embolization.^27^ A randomized clinical trial demonstrated superior success rates for hysteroscopic resection compared with those for ultrasound-guided S&C^28^ However, hysteroscopy or any other intrauterine surgical intervention should only be employed when myometrial thickness is ⩾2 mm and the gestational mass measures <3 cm.^25^ Others suggest a minimum uninvolved myometrial thickness of ⩾3 mm.^26^ According to a Cochrane review, S&C—especially when combined with uterine artery embolization—remains a safe alternative, and the addition of hysteroscopy does not significantly improve treatment success (RR 0.91; 95% CI: 0.81–1.03).^29^

A meta-analysis identified multiparity, large gestational sac size, advanced gestational age, elevated serum hCG levels, abundant vascularization of the gestational sac, and a thin myometrial layer as significant risk factors for massive hemorrhage during the management of CSP.^30^ Several authors have suggested the adjunctive use of rectal misoprostol, in addition to uterine artery embolization, to further reduce the risk of hemorrhage.^14^ Administration of MTX and mifepristone can be considered an effective first-line alternative treatment for CSP, especially in cases with hCG levels below 5000 IU/L and a gestational sac diameter of less than 20 mm.^31^ However, current guidelines advise against the use of systemic MTX as a sole therapeutic modality and instead recommend early termination using local MTX or surgical approaches, accompanied by strict follow-up until complete resolution.^32^

A more recent conservative strategy involves the use of a double-balloon catheter placed in the CSP area.^33^ Observational management may be considered for endogenous type I CSP in the absence of symptoms. A systematic review of 28 studies reported that 78% of patients with CSP who underwent observational management achieved live birth at or near term.^34^ However, this approach was associated with substantial morbidity, with 55% requiring hysterectomy and 40% experiencing severe hemorrhage.^34,35^ Additionally, three or more previous cesarean deliveries, mass-type CSP, serum hCG levels exceeding 20,000 IU/L, and an interval of more than 3 years since the last cesarean section were identified as significant risk factors for the failure of expectant management.^36^ Furthermore, Society for Maternal-Fetal Medicine (SMFM) guidelines also recommend against observational management (evidence-based medicine (EBM) grade 1B).^37^

The management options for cervical ectopic pregnancy are largely similar to those used for CSP, although the risk of major hemorrhage is higher, and hysterectomy may be required in severe cases. Other types of ectopic pregnancy are treated primarily with pharmacologic therapy using MTX or surgical intervention. Interstitial ectopic pregnancy may be managed with conservative surgical techniques such as cornuotomy or wedge resection, preferably performed laparoscopically. Ovarian ectopic pregnancy is most often diagnosed intraoperatively and treated surgically, typically through ovarian wedge resection or cystectomy, with oophorectomy reserved for cases of uncontrolled bleeding. Abdominal ectopic pregnancy is managed predominantly by surgical removal via laparotomy, and placental management—whether removal or leaving it in situ—is a critical determinant of maternal morbidity due to the substantial risk of life-threatening hemorrhage.^1,4,5^ Non-tubal ectopic pregnancies, in particular, are potentially catastrophic and associated with severe morbidity if not recognized early. Consequently, some experts advocate for the involvement of reproductive endocrinology and infertility specialists in their diagnosis and management to help standardize care and improve outcomes for these high-risk pregnancies.^38^

### Reproductive outcomes

A previous ectopic pregnancy is associated with an increased risk of several adverse perinatal outcomes in subsequent pregnancies, most prominently recurrent ectopic pregnancy, with almost twice the risk (odds ratio (OR) 1.97). Elevated risks have also been reported for preterm birth, placental abruption, hypertensive disorders, low birth weight, and emergency cesarean delivery.^39^ Nonetheless, the odds of subsequent IUP (OR 1.25) and recurrent ectopic pregnancy (OR 0.69) do not differ significantly between women treated with MTX and those managed expectantly for tubal pregnancy.^40^ In a retrospective cohort study, expectant management was associated with the most favorable reproductive outcomes—including the highest IUP rate (65.3%, *p* = 0.012), the highest live birth rate (*p* = 0.035), and the shortest time to conception (*p* < 0.05)—while the risk of recurrent ectopic pregnancy remained similar across treatment groups.^41^

To improve future fertility, Bruhat et al. introduced the tubal-sparing approach of laparoscopic salpingotomy in the 1980s.^42^ Multiple meta-analyses have supported this strategy. A meta-analysis of 1591 participants showed significantly higher odds of IUP after salpingostomy than after salpingectomy (pooled OR 1.61, *p* < 0.001).^40^ A meta-analysis of 15 randomized controlled trials (RCTs) similarly demonstrated a significantly greater IUP rate following salpingotomy (OR 2.49, *p* < 0.0001),^43^ and an analysis of 11 studies reported a pooled IUP rate of 61.2% after salpingotomy (*p* = 0.000).^44^

However, other studies have not confirmed a fertility advantage for salpingotomy.^45–47^ A recent retrospective study from Central Europe reported significantly more recurrent ectopic pregnancies after salpingotomy (*p* = 0.029), with no significant difference in live birth rates (*p* = 0.201); women undergoing salpingotomy also had significantly smaller ectopic masses (*p* < 0.001). A meta-analysis of two RCTs found no significant difference in spontaneous IUP rates between salpingotomy and salpingectomy (RR 1.04, *p* = 0.61), whereas eight cohort studies demonstrated significantly higher spontaneous IUP rates after salpingotomy (RR 1.24, *p* = 0.002).^47^ Similarly, another meta-analysis found no difference in RCTs (OR 0.97), but cohort studies showed a markedly lower likelihood of IUP after salpingectomy (OR 0.45).^46^ These conflicting results likely reflect differing selection criteria, as salpingectomy in cohort studies was usually performed in women with larger ectopic masses, severely damaged tubes, or older age with reduced fertility potential.^46,47^ Higher IUP rates after salpingotomy were also more frequently reported in studies conducted before 2020 (*p* = 0.013) and among women younger than 30 years (*p* = 0.007).^44^

CSP is the most common non-tubal ectopic pregnancy. A meta-analysis of 32 studies reported a 76.2% fertility rate among women attempting conception after CSP treatment, with 83.4% achieving an IUP and 16.6% experiencing an ectopic pregnancy—most frequently recurrent CSP (15.3%).^48^ Another meta-analysis of 44 studies including 3598 women with prior CSP documented miscarriage, preterm birth, and placenta accreta spectrum disorders in 19.1%, 10.3%, and 4.0% of pregnancies, respectively, with a higher incidence in women treated surgically rather than nonsurgically.^49^ Overall, most women with a history of CSP retain good reproductive potential, with 70%–76% eventually conceiving and over 80% achieving an IUP. However, subsequent pregnancies are characterized by substantial risk—including recurrent CSP (15%–34%), miscarriage (15%–20%), preterm birth (~10%), placenta accreta spectrum disorders (~4%), and uterine rupture (~1%–2%).^50^ To date, there is no convincing evidence that any specific treatment modality (medical versus surgical; scar repair versus no repair) effectively mitigates these risks.^48,49^

### Aim of the survey

The aim of this nationwide cross-sectional, observational, questionnaire-based study was to evaluate the availability, frequency of use, and institutional variability of conservative, medical, and surgical management options for ectopic pregnancy across all inpatient gynecological departments, and to determine how treatment patterns differ according to institutional size, defined by annual surgical volume.

## Materials and methods

**Figure 2.**
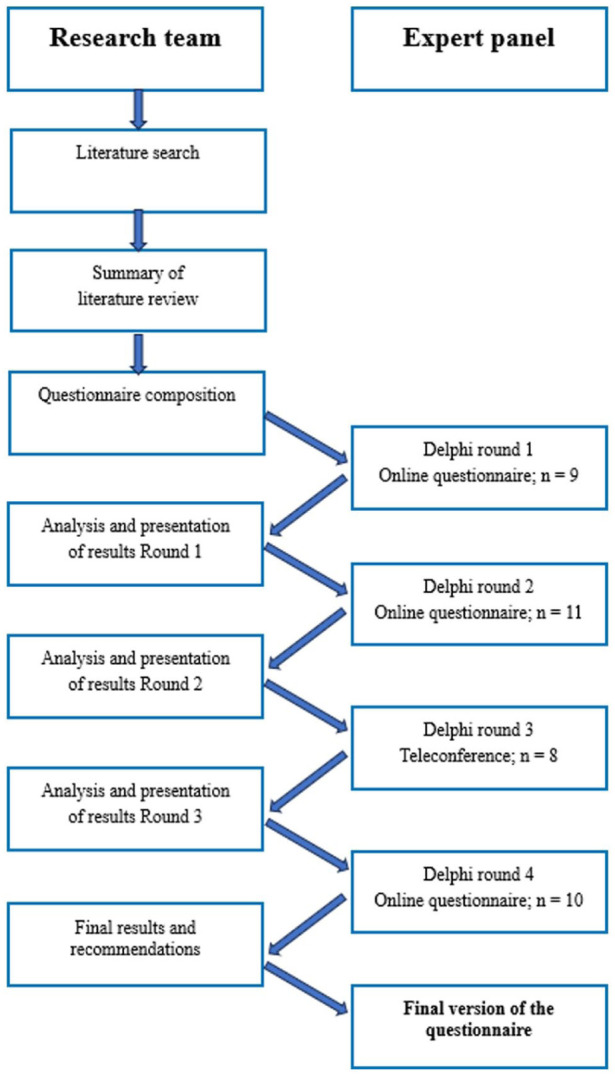
Study design: stepwise modified Delphi method used to achieve consensus on the questionnaire regarding ectopic pregnancy management and treatment strategies.

This observational cross-sectional questionnaire study adhered to the relevant reporting guidelines and the STROBE checklist is provided as Supplemental Table S1.^51^ The literature review and consensus-building process took place between July and December 2024. A modified four-round Delphi method was used to achieve expert agreement (Figure 2).^52^ A comprehensive search of MEDLINE, CENTRAL, PubMed, and EMBASE was conducted for January 2014–June 2024 using the terms “ectopic pregnancy,” “tubal pregnancy,” “cesarean scar pregnancy,” “cervical pregnancy,” “interstitial pregnancy,” and “pregnancy of unknown location.” Only English-language articles addressing definitions, diagnosis, and management of ectopic pregnancy were reviewed.

Based on these data, a questionnaire was developed covering diagnostic and therapeutic approaches for tubal pregnancy, CSP, cervical pregnancy, interstitial pregnancy, and PUL. Standardized terminology was applied throughout. Eleven gynecologic surgeons with ⩾10 years of experience managing acute gynecologic conditions, including ectopic pregnancy, were invited to participate.

The Delphi procedure consisted of repeated questionnaire rounds. After each round, responses were analyzed, and unresolved items were carried forward with anonymized feedback. The process included three online rounds and one teleconference, with consensus defined as ⩾70% agreement. Clarification questions and new items were added as needed. A preliminary set of recommendations was drafted after the second round. Items reaching consensus were reviewed again, and several required clarification in a fourth digital round. The final agreed-upon items were circulated for approval. Minor revisions requested during peer review were incorporated and approved by all experts.

Although formal psychometric validation was not performed, the preliminary version of the questionnaire was pilot-tested in a prior nationwide questionnaire study evaluating the management of ectopic pregnancy in the Czech Republic. This pilot study included 45 of 95 inpatient gynecological departments (47.9% of the national target population) and was published in a peer-reviewed journal.^53^ Feedback obtained during this pilot testing was used to refine the questionnaire items, response options, and clarity prior to implementation in the present study.

The types of ectopic pregnancies were classified according to the recommendations of ESHRE (the European Society of Human Reproduction and Embryology).^4,7^ The questionnaire included items addressing the management of the more common types of ectopic pregnancy, such as tubal pregnancy (implantation within the fallopian tube), CSP (implantation in the anterior lower uterine segment at the site of a previous cesarean-section scar), cervical pregnancy (implantation within the cervical tissue below the level of the internal os), interstitial ectopic pregnancy (implantation in the interstitial, intramural segment of the fallopian tube at the uterine cornua), as well as PUL. Questions regarding rare forms of ectopic pregnancy—such as ovarian, abdominal, rudimentary horn, and intramural pregnancies—were not included in the questionnaire. Some questions were designed as multiple-choice items.

In January 2025, the Ministry of Health of the Czech Republic registered 92 inpatient healthcare facilities providing 24-h acute gynecological care. We contacted the heads of all of 92 departments by phone, inviting them to anonymously complete an online questionnaire focused on the management of different types of ectopic pregnancy. The questionnaire (Supplemental Table S2) was created using the Google Forms web platform (forms.google.com), and a link to the survey was subsequently distributed via email on January 21, 2025. By February 9, 46 responses were received (51.7%). Between 10 and 14 February, we contacted nonresponding departments by telephone, yielding an additional 39 questionnaires by 2 March (92.4%). A final round of reminders between 3 and 7 March resulted in four more responses, achieving a final response rate of 96.7% (89/92).

This study was a questionnaire-based, institution-level survey and did not involve patients, biological material, or the collection of personal or identifiable data. The questionnaire addressed departmental diagnostic and therapeutic practices only. Participation was voluntary, anonymous, and implied consent at the institutional level. According to Czech national legislation (Act No. 110/2019 Coll., on the Processing of Personal Data, implementing the General Data Protection Regulation) and institutional regulations, ethics committee or institutional review board (IRB) approval is not required for fully anonymized surveys that do not involve human subjects.^54^ Therefore, formal IRB approval was not sought.

### Inclusion criteria

Ninety-two inpatient health are facilities providing 24-h acute gynecological care, as registered by the Ministry of Health of the Czech Republic in January 2025.

### Exclusion criteria

Healthcare facilities that did not complete the online questionnaire distributed for the study.

### Statistical analysis

Institutions were categorized into four groups according to their total annual number of hysterectomies: Group S (small, ⩽100 hysterectomies/year (hpy)), Group M (medium, 101–200 hpy), Group L (large, 201–300 hpy), and Group X (extra large, >300 hpy). Missing data were handled by pairwise deletion, meaning that analyses were performed using all available responses for each individual questionnaire item. No imputation methods were applied, as the questionnaire pertained to institutional practices rather than to patient-level data, and the pattern of missingness was judged to be random.

All variables in the dataset were categorical. Consequently, we used Fisher’s exact test with Monte Carlo simulation (10,000 iterations) to allow valid inference in tables with small cell counts or sparse data, which are typical in surveys involving rare conditions such as cervical or CSP. For overall comparisons across the four institutional volume groups, as well as for subgroup comparisons between smaller (S/M) and larger (L/X) centers, Fisher’s test was the most appropriate nonparametric method. To account for multiple comparisons across questionnaire items, we applied false discovery rate adjustment, reporting both *p*-values and *q*-values. A two-sided *p*-value <0.05, along with the corresponding *q*-value, was considered statistically significant. All analyses were performed using R (version 4.4.3), consistent with standard practice for categorical survey data.^55,56^

This study was designed as a nationwide census survey. All inpatient gynecological departments providing 24-h acute gynecological care in the Czech Republic (*n* = 92), as registered by the Ministry of Health in January 2025, were invited to participate. The aim was to capture the full national spectrum of institutional practices rather than to draw inferences from a predefined sample. Consequently, no formal sample size calculation or power analysis was performed.

## Results

A total of 89 out of 92 eligible gynecological departments in the Czech Republic that provide 24/7 emergency gynecological care completed the anonymous questionnaire, corresponding to a response rate of 96.7%. Sixteen institutions (18.0%) reported performing ⩽100 hysterectomies per year (Group S), 44 (49.4%) performed 101–200 (Group M), 21 (23.6%) performed 201–300 (Group L), and 8 (9.0%) performed >300 procedures annually (Group X). The basic characteristics of the responding institutions are summarized in Table 1. A maternity unit was part of 81 (91%) of the surveyed departments. As expected, institutions performing higher volumes of hysterectomies were more often located in larger municipalities, held higher levels of national accreditation, and reported greater numbers of annual deliveries. Table 2 presents a summary of the findings stratified by institutional volume (Groups S, M, L, and X), while Table 3 compares the responses of smaller (Group S/M, ⩽200 hpy) versus larger institutions (Group L/X, >200 hpy).

**Table 1.** Basic characteristics of the participating facilities.

Characteristics	All departments (*N* = 89)	Group S (small) ⩽100 hpy, *N* = 16 (18.0%)	Group M (medium) 101–200 hpy, *N* = 44 (49.4%)	Group L (large) 201–300 hpy, *N* = 21 (23.6%)	Group X (extra large) >300 hpy, *N* = 8 (9.0%)	*p*-value	*q*-value
Population of the municipality where the facility is based							
<10,000	3 (3.4%)	0 (0.0%)	3 (6.8%)	0 (0.0%)	0 (0.0%)	<0.001	0.004
10,001–20,000	20 (22.5%)	6 (37.5%)	11 (25.0%)	3 (14.3%)	0 (0.0%)		
20,001–30,000	19 (21.3%)	6 (37.5%)	11 (25.0%)	2 (9.5%)	0 (0.0%)		
30,001–50,000	17 (19.1%)	2 (12.5%)	11 (25.0%)	4 (19.0%)	0 (0.0%)		
50,001–100,000	12 (13.5%)	0 (0.0%)	7 (15.9%)	3 (14.3%)	2 (25.0%)		
100,001–1,000,000	9 (10.1%)	1 (6.3%)	1 (2.3%)	4 (19.0%)	3 (37.5%)		
>1,000,000	9 (10.1%)	1 (6.3%)	0 (0.0%)	5 (23.8%)	3 (37.5%)		
State accreditation of the gynecological department							
Without accreditation	10 (11.2%)	7 (43.8%)	3 (6.8%)	0 (0.0%)	0 (0.0%)	<0.001	0.003
Accreditation type I	54 (60.7%)	7 (43.8%)	36 (81.8%)	10 (47.6%)	1 (12.5%)		
Accreditation type II	14 (15.7%)	2 (12.5%)	3 (6.8%)	7 (33.3%)	2 (25.0%)		
Accreditation type III	11 (12.4%)	0 (0.0%)	2 (4.5%)	4 (19.0%)	5 (62.5%)		
Number of deliveries in 2023							
No maternity hospital	7 (7.9%)	4 (25.0%)	1 (2.3%)	2 (9.5%)	0 (0.0%)	<0.001	0.003
⩽600	17 (19.1%)	6 (37.5%)	11 (25.0%)	0 (0.0%)	0 (0.0%)		
601–1000	37 (41.6%)	5 (31.3%)	22 (50.0%)	9 (42.9%)	1 (12.5%)		
1001–2000	19 (21.3%)	1 (6.3%)	8 (18.2%)	7 (33.3%)	3 (37.5%)		
2001–3000	6 (6.7%)	0 (0.0%)	2 (4.5%)	3 (14.3%)	1 (12.5%)		
>3000	3 (3.4%)	0 (0.0%)	0 (0.0%)	0 (0.0%)	3 (37.5%)		
Number of deliveries in 2024							
No maternity hospital	8 (9.0%)	4 (25.0%)	2 (4.5%)	2 (9.5%)	0 (0.0%)	<0.001	0.004
<600	25 (28.1%)	6 (37.5%)	17 (38.6%)	2 (9.5%)	0 (0.0%)		
601–1000	33 (37.1%)	6 (37.5%)	18 (40.9%)	8 (38.1%)	1 (12.5%)		
1001–2000	16 (18.0%)	0 (0.0%)	7 (15.9%)	6 (28.6%)	3 (37.5%)		
2001–3000	4 (4.5%)	0 (0.0%)	0 (0.0%)	3 (14.3%)	1 (12.5%)		
>3000	3 (3.4%)	0 (0.0%)	0 (0.0%)	0 (0.0%)	3 (37.5%)		
Availability of chemotherapy in a medical facility							
Yes	47 (52.8%)	5 (31.3%)	23 (52.3%)	13 (61.9%)	6 (75.0%)	0.2	0.3
No	42 (47.2%)	11 (68.8%)	21 (47.7%)	8 (38.1%)	2 (25.0%)		
Common availability of methotrexate in a medical facility							
Yes	53 (59.6%)	4 (25.0%)	25 (56.8%)	18 (85.7%)	6 (75.0%)	0.002	0.015
No	36 (40.4%)	12 (75.0%)	19 (43.2%)	3 (14.3%)	2 (25.0%)		

hpy: hysterectomies per year.

**Table 2.** Results by department size.

Ectopic pregnancy management	All departments (*N* = 89)	Group S (small) ⩽100 hpy, *N* = 16 (18.0%)	Group M (medium) 101–200 hpy, *N* = 44 (49.4%)	Group L (large) 201–300 hpy, *N* = 21 (23.6%)	Group X (extra large) >300 hpy, *N* = 8 (9.0%)	*p*-value	*q*-value
MTX section							
Methotrexate use for ectopic pregnancy treatment							
Yes, commonly	16 (18.0%)	2 (12.5%)	7 (15.9%)	5 (23.8%)	2 (25.0%)	0.3	0.5
Atypical site only	32 (35.9%)	5 (31.3%)	13 (29.5%)	11 (52.4%)	3 (37.5%)		
Never	41 (46.1%)	9 (56.3%)	24 (54.5%)	5 (23.8%)	3 (37.5%)		
Method of methotrexate administration							
Topical (locally) only	4 (4.5%)	0 (0.0%)	1 (2.3%)	2 (9.5%)	1 (12.5%)	0.2	0.4
Systemic (i.m.) only	33 (37.1%)	6 (37.5%)	14 (31.8%)	9 (42.9%)	4 (50.0%)		
Systemic + topical	11 (12.3%)	1 (6.3%)	5 (11.4%)	5 (23.8%)	0 (0.0%)		
Never	41 (46.1%)	9 (56.3%)	24 (54.5%)	5 (23.8%)	3 (37.5%)		
Physician-recommended interval before attempting conception after completion of methotrexate therapy							
Whenever	2 (2.3%)	1 (6.3%)	0 (0.0%)	1 (4.8%)	0 (0.0%)	0.7	0.8
In 3 months	23 (25.8%)	4 (25.0%)	11 (25.0%)	6 (28.6%)	2 (25.0%)		
In 6 months	64 (71.9%)	11 (68.8%)	33 (75.0%)	14 (66.7%)	6 (75.0%)		
Tubal pregnancy section							
General management approach for intact tubal pregnancy							
Always surgery	32 (36.0%)	6 (37.5%)	14 (31.8%)	7 (33.3%)	5 (62.5%)	0.3	0.5
Sometimes nonsurgical management (observation, MTX)	57 (64.0%)	10 (62.5%)	30 (68.2%)	14 (66.7%)	3 (37.5%)		
Clinical criteria for methotrexate administration in cases of tubal pregnancy							
Never	73 (82.0%)	14 (87.5%)	37 (84.1%)	16 (76.2%)	6 (75.0%)	0.4	0.6
hCG level only	12 (13.5%)	0 (0.0%)	3 (6.8%)	0 (0.0%)	1 (12.5%)		
hCG level + US finding	4 (4.5%)	2 (12.5%)	4 (9.1%)	5 (23.8%)	1 (12.5%)		
Frequency of methotrexate use in the management of tubal pregnancy							
Never	73 (82.0%)	14 (87.5%)	37 (84.1%)	16 (76.2%)	6 (75.0%)	0.6	0.8
⩽5%	8 (9.0%)	1 (6.3%)	5 (11.4%)	1 (4.8%)	1 (12.5%)		
5%–20%	7 (7.9%)	1 (6.3%)	2 (4.5%)	3 (14.3%)	1 (12.5%)		
>20%	1 (1.1%)	0 (0.0%)	0 (0.0%)	1 (4.8%)	0 (0.0%)		
Surgical approaches for intact tubal pregnancy							
Always salpingectomy	55 (61.8%)	8 (50.0%)	28 (63.6%)	14 (66.7%)	5 (62.5%)	0.7	0.8
Sometimes salpingostomy	34 (38.2%)	8 (50.0%)	16 (36.4%)	7 (33.3%)	3 (37.5%)		
D&C during surgical treatment of tubal pregnancy							
Never	15 (16.8%)	1 (6.3%)	6 (13.6%)	4 (19.0%)	4 (50.0%)	0.041	0.13
Sometimes	66 (74.2%)	11 (68.8%)	34 (77.3%)	17 (81.0%)	4 (50.0%)		
Always	8 (9.0%)	4 (25.0%)	4 (9.1%)	0 (0.0%)	0 (0.0%)		
Interstitial pregnancy section							
Preferred approaches for the management of diagnosed interstitial pregnancy (multiple responses permitted)							
No experience/transfer the case to another department	7 (7.9%)	2 (12.5%)	5 (11.4%)	0 (0.0%)	0 (0.0%)	0.3	0.6
Systemic MTX i.m. administration	13 (14.6%)	1 (6.3%)	7 (15.9%)	5 (23.8%)	0 (0.0%)	0.4	0.6
Local MTX administration	7 (7.9%)	2 (12.5%)	2 (4.5%)	3 (14.3%)	0 (0.0%)	0.4	0.6
Laparoscopic surgery	77 (86.5%)	11 (68.8%)	37 (84.1%)	21 (100.0%)	8 (100.0%)	0.023	0.08
Laparotomic surgery	35 (39.3%)	10 (62.5%)	16 (36.4%)	6 (28.6%)	3 (37.5%)	0.2	0.4
Performance of salpingectomy during surgical management of interstitial pregnancy							
No experience	7 (7.9%)	2 (12.5%)	5 (11.4%)	0 (0.0%)	0 (0.0%)	0.004	0.026
Always salpingectomy	64 (71.9%)	13 (81.3%)	34 (77.3%)	10 (47.6%)	7 (87.5%)		
Sometimes tube preservation	18 (20.2%)	1 (6.3%)	5 (11.4%)	11 (52.4%)	1 (12.5%)		
CSP section							
Number of CSP cases diagnosed in the department over the past 5 years							
0	21 (23.6%)	7 (43.8%)	8 (18.2%)	6 (28.6%)	0 (0.0%)	0.003	0.022
1–2	34 (38.2%)	5 (31.3%)	24 (54.5%)	4 (19.0%)	1 (12.5%)		
⩾3	34 (38.2%)	4 (25.0%)	12 (27.3%)	11 (52.4%)	7 (87.5%)	____________________	
Possibility of observation-only approach in CSP							
Yes	6 (6.7%)	1 (6.3%)	3 (6.8%)	2 (9.5%)	0 (0.0%)	>0.9	>0.9
No	83 (93.3%)	15 (93.8%)	41 (93.2%)	19 (90.5%)	8 (100.0%)		
Preferred approaches for the management of diagnosed CSP (multiple responses permitted)							
No experience/transfer the case to another department	28 (31.5%)	11 (68.8%)	14 (31.8%)	3 (14.3%)	0 (0.0%)	<0.001	0.007
Expectation/observation	0 (0.0%)	0 (0.0%)	0 (0.0%)	0 (0.0%)	0 (0.0%)	>0.9	>0.9
Systemic MTX administration (i.m.)	18 (20.2%)	3 (18.8%)	8 (18.2%)	6 (28.6%)	1 (12.5%)	0.7	0.8
Local MTX administration	9 (10.1%)	2 (12.5%)	3 (6.8%)	3 (14.3%)	1 (12.5%)	0.6	0.8
Misoprostol administration (vaginally, rectally)	10 (11.2%)	1 (6.3%)	6 (13.6%)	3 (14.3%)	0 (0.0%)	0.8	0.8
S&C	42 (47.2%)	3 (18.8%)	19 (43.2%)	13 (61.9%)	7 (87.5%)	0.006	0.029
Hysteroscopic resection	23 (25.8%)	1 (6.3%)	11 (25.0%)	9 (42.9%)	2 (25.0%)	0.083	0.2
Insertion of a balloon into the cervical canal for several days	1 (1.1%)	0 (0.0%)	0 (0.0%)	1 (4.8%)	0 (0.0%)	0.5	0.7
Laparoscopic surgery	5 (5.6%)	0 (0.0%)	2 (4.5%)	2 (9.5%)	1 (12.5%)	0.4	0.6
Laparoscopic or laparotomic surgery	14 (15.7%)	0 (0.0%)	8 (18.2%)	5 (23.8%)	1 (12.5%)	0.2	0.4
Combination of laparoscopic and vaginal approach	21 (23.6%)	0 (0.0%)	10 (22.7%)	7 (33.3%)	4 (50.0%)	0.015	0.062
Hysterectomy performed for CSP in the past 5 years							
Yes	3 (3.4%)	0 (0.0%)	0 (0.0%)	2 (9.5%)	1 (12.5%)	0.06	0.2
No	86 (96.6%)	16 (100.0%)	44 (100.0%)	19 (90.5%)	7 (87.5%)		
Cervical pregnancy section (not CSP)							
Number of cervical pregnancy cases diagnosed in the department over the past 5 years							
0	51 (57.3%)	13 (81.3%)	30 (68.2%)	7 (33.3%)	1 (12.5%)	<0.001	0.004
1	33 (37.1%)	3 (18.8%)	13 (29.5%)	13 (61.9%)	4 (50.0%)		
⩾ 2	5 (5.6%)	0 (0.0%)	1 (2.3%)	1 (4.8%)	3 (37.5%)		
Possibility of observation-only approach in cervical pregnancy							
Yes	6 (6.7%)	1 (6.3%)	3 (6.8%)	2 (9.5%)	0 (0.0%)	>0.9	>0.9
No	83 (93.3%)	15 (93.8%)	41 (93.2%)	19 (90.5%)	8 (100.0%)		
Preferred approaches for the management of diagnosed cervical pregnancy (multiple responses permitted)							
No experience/transfer the case to another department	26 (23.1%)	10 (62.5%)	12 (27.3%)	4 (19.0%)	0 (0.0%)	0.006	0.029
Systemic MTX i.m. administration	23 (25.8%)	2 (12.5%)	9 (20.5%)	9 (42.9%)	3 (37.5%)	0.12	0.3
Local MTX administration	15 (16.9%)	1 (6.3%)	5 (11.4%)	6 (28.6%)	3 (37.5%)	0.078	0.2
Misoprostol administration (vaginally, rectally)	15 (16.9%)	1 (6.3%)	9 (20.5%)	4 (19.0%)	1 (12.5%)	0.7	0.8
S&C	54 (60.7%)	5 (31.3%)	27 (61.4%)	15 (71.4%)	7 (87.5%)	0.032	0.11
Hysteroscopic resection	17 (19.1%)	2 (12.5%)	8 (18.2%)	6 (28.6%)	1 (12.5%)	0.7	0.7
Insertion of a balloon into the cervical canal for several days	1 (1.1%)	0 (0.0%)	0 (0.0%)	1 (4.8%)	0 (0.0%)	0.5	0.7
Laparoscopic surgery	1 (1.1%)	0 (0.0%)	0 (0.0%)	1 (4.8%)	0 (0.0%)	0.5	0.7
Combination of laparoscopic and vaginal approach	4 (4.5%)	0 (0.0%)	3 (6.8%)	0 (0.0%)	1 (12.5%)	0.2	0.4
Laparoscopic or laparotomic surgery	9 (10.1%)	0 (0.0%)	6 (13.6%)	2 (9.5%)	1 (12.5%)	0.5	0.7
Hysterectomy performed for cervical pregnancy in the past 5 years							
Yes	2 (2.3%)	0 (0.0%)	1 (2.3%)	1 (4.8%)	0 (0.0%)	0.8	0.8
No	87 (97.7%)	16 (100.0%)	43 (97.7%)	20 (95.2%)	8 (100.0%)		
Clinical practice of uterine artery ligation for hemorrhage control in cases of large cervical or CSP							
Yes	13 (14.6%)	0 (0.0%)	5 (11.4%)	5 (23.8%)	3 (37.5%)	0.033	0.11
No	76 (85.4%)	16 (100.0%)	39 (88.6%)	16 (76.2%)	5 (62.5%)		
PUL section							
Preferred approach to PUL with falling hCG levels							
No experience	0 (0.0%)	0 (0.0%)	0 (0.0%)	0 (0.0%)	0 (0.0%)	>0.9	>0.9
Expectation/observation	74 (83.2%)	9 (56.3%)	36 (81.8%)	21 (100.0%)	8 (100.0%)	0.006	0.029
RCUI	13 (14.6%)	7 (43.8%)	6 (13.6%)	0 (0.0%)	0 (0.0%)		
Diagnostic laparoscopy	2 (2.2%)	0 (0.0%)	2 (4.5%)	0 (0.0%)	0 (0.0%)		
Systemic MTX i.m. administration	0 (0.0%)	0 (0.0%)	0 (0.0%)	0 (0.0%)	0 (0.0%)		
Preferred approach to PUL with not falling hCG levels							
No experience/transfer the case to another department	4 (4.5%)	1 (6.3%)	3 (6.8%)	0 (0.0%)	0 (0.0%)	0.7	0.8
Expectation/observation	4 (4.5%)	1 (6.3%)	1 (2.3%)	2 (9.5%)	0 (0.0%)		
RCUI	21 (23.6%)	3 (18.8%)	11 (25.0%)	5 (23.8%)	2 (25.0%)		
Diagnostic laparoscopy	42 (47.2%)	9 (56.3%)	21 (47.7%)	8 (38.1%)	4 (50.0%)		
RCUI + diagnostic laparoscopy	9 (10.1%)	1 (6.3%)	6 (13.6%)	1 (4.8%)	1 (12.5%)		
Systemic MTX i.m. administration	9 (10.1%)	1 (6.3%)	2 (4.5%)	5 (23.8%)	1 (12.5%)		

hpy: hysterectomies per year; MTX: methotrexate; hCG: human chorionic gonadotropin; RCUI: revisio cavi uteri instrumentalis; D&C: dilation and curettage; CSP: cesarean scar pregnancy; i.m.: intramuscular injection; S&C: suction and curettage; PUL: pregnancy of unknown location; US: ultrasound.

**Table 3.** Comparison of selected results by department size performing ⩾200 versus <200 hysterectomies per year.

Ectopic pregnancy management	All departments (*N* = 89)	Group S/M (small/medium) ⩽200 hpy, *N* = 60 (67.4%)	Group L/X (large/extra large) >200 hpy, *N* = 29 (32.6%)	*p*-value	*q*-value
MTX section					
Common availability of methotrexate in a medical facility					
Yes	53 (59.6%)	29 (48.3%)	24 (82.8%)	0.002	0.02
No	36 (40.4%)	31 (51.7%)	5 (17.2%)		
Methotrexate use for ectopic pregnancy treatment					
Yes	48 (53.9%)	27 (45.0%)	21 (72.4%)	0.023	0.086
No	41 (46.1%)	33 (55.0%)	8 (27.6%)		
Methotrexate use for ectopic pregnancy treatment					
Yes, commonly	16 (18.0%)	9 (15.0%)	7 (24.1%)	0.05	0.13
Atypical site only	32 (35.9%)	18 (30.0%)	14 (48.3%)		
Never	41 (46.1%)	33 (55.0%)	8 (27.6%)		
Method of methotrexate administration					
Systemic (i.m.) only	33 (37.1%)	20 (33.3%)	13 (44.8%)	0.03	0.095
Systemic and/or Topical	15 (16.9%)	7 (11.7%)	8 (27.6%)		
Never	41 (46.1%)	33 (55.0%)	8 (27.6%)		
Physician-recommended interval before attempting conception after the completion of methotrexate therapy					
In 6 months	64 (71.9%)	44 (73.3%)	20 (69.0%)	0.8	>0.9
Earlier	25 (28.1%)	16 (26.7%)	9 (31.0%)		
Tubal pregnancy section					
General management approach for intact tubal pregnancy					
Always surgery	32 (36.0%)	20 (33.3%)	12 (41.4%)	0.5	0.6
Sometimes nonsurgical management (observation, MTX)	57 (64.0%)	40 (66.7%)	17 (58.6%)		
Methotrexate use in the management of tubal pregnancy					
Yes	16 (18.0%)	9 (15.0%)	7 (24.1%)	0.4	0.5
No	73 (82.0%)	51 (85.0%)	22 (75.9%)		
Surgical approaches for intact tubal pregnancy					
Always salpingectomy	55 (61.8%)	36 (60.0%)	19 (65.5%)	0.6	0.8
Sometimes salpingostomy	34 (38.2%)	24 (40.0%)	10 (34.5%)		
D&C during surgical treatment of tubal pregnancy					
Never	15 (16.8%)	7 (11.7%)	8 (27.6%)	0.021	0.085
Sometimes	66 (74.2%)	45 (75.0%)	21 (72.4%)		
Always	8 (9.0%)	8 (13.3%)	0 (0.0%)		
Interstitial pregnancy section					
Preferred approaches for the management of diagnosed interstitial pregnancy (multiple responses permitted)					
No experience/transfer the case to another department	7 (7.9%)	7 (11.7%)	0 (0.0%)	0.091	0.2
Systemic MTX i.m. administration	13 (14.6%)	8 (13.3%)	5 (17.2%)	0.8	0.9
Local MTX administration	7 (7.9%)	4 (6.7%)	3 (10.3%)	0.7	0.8
Laparoscopic surgery	77 (86.5%)	48 (80.0%)	29 (100.0%)	0.007	0.036
Laparotomic surgery	35 (39.3%)	26 (43.3%)	9 (31.0%)	0.2	0.4
Preferred approaches for the management of diagnosed interstitial pregnancy (multiple responses permitted)					
No experience	7 (7.9%)	7 (11.7%)	0 (0.0%)	0.091	0.2
MTX treatment	17 (19.1%)	10 (16.7%)	7 (24.1%)	0.4	0.5
Peritoneal surgery	82 (92.1%)	53 (88.3%)	29 (100.0%)	0.091	0.15
Performance of salpingectomy during surgical management of interstitial pregnancy					
No experience	7 (7.9%)	7 (11.7%)	0 (0.0%)	0.091	0.2
Always salpingectomy	64 (71.9%)	47 (88.7%)	17 (58.6%)	0.004	0.023
Sometimes tube preservation	18 (20.2%)	6 (11.3%)	12 (41.4%)		
CSP section					
Number of CSP cases diagnosed in the department over the past 5 years					
0	21 (23.6%)	15 (25.0%)	6 (20.7%)	0.003	0.021
1–2	34 (38.2%)	29 (48.3%)	5 (17.2%)		
⩾ 3	34 (38.2%)	16 (26.7%)	18 (62.1%)		
Possibility of observation-only approach in CSP					
Yes	7 (7.9%)	5 (8.3%)	2 (6.9%)	>0.9	>0.9
No	82 (92.1%)	55 (91.7%)	27 (93.1%)		
Preferred approaches for the management of diagnosed CSP (multiple responses permitted)					
Transfer the case to another department/no experience	28 (31.5%)	25 (41.7%)	3 (10.3%)	0.003	0.021
Systemic application (i.m.) of MTX	18 (20.2%)	11 (18.3%)	7 (24.1%)	0.6	0.7
Local application of MTX	9 (10.1%)	5 (8.3%)	4 (13.8%)	0.5	0.6
Misoprostol administration (vaginally, rectally)	10 (11.2%)	7 (11.7%)	3 (10.3%)	>0.9	>0.9
S&C	42 (47.2%)	22 (36.7%)	20 (69.0%)	0.006	0.033
Hysteroscopic resection	23 (25.8%)	12 (20.0%)	11 (37.9%)	0.078	0.2
Insertion of a balloon into the cervical canal for several days	1 (1.1%)	0 (0.0%)	1 (3.4%)	0.3	0.5
Laparoscopic surgery	5 (5.6%)	2 (3.3%)	3 (10.3%)	0.3	0.5
Combination of laparoscopic and vaginal approach	21 (23.6%)	10 (16.7%)	11 (37.9%)	0.035	0.1
Laparotomic surgery	2 (2.2%)	1 (1.7%)	1 (3.4%)	0.5	0.7
Peritoneal (laparoscopic or laparotomic) surgery	14 (15.7%)	8 (13.3%)	6 (20.7%)	0.4	0.5
Preferred approaches for the management of diagnosed CSP (multiple responses permitted)					
No experience	28 (31.5%)	25 (41.7%)	3 (10.3%)	0.003	0.021
Pharmacological treatment	28 (31.5%)	16 (26.7%)	12 (41.4%)	0.2	0.3
Vaginal surgery	48 (53.9%)	26 (43.3%)	22 (75.9%)	0.006	0.020
Peritoneal surgery	33 (37.1%)	15 (25.0%)	18 (62.1%)	0.001	0.006
Hysterectomy performed for CSP in the past 5 years					
Yes	3 (3.4%)	0 (0.0%)	3 (10.3%)	0.032	0.1
No	86 (96.6%)	60 (100.0%)	26 (89.7%)		
Cervical pregnancy section (not CSP)					
Number of cervical pregnancy cases diagnosed in the department over the past 5 years					
0	51 (57.3%)	43 (71.7%)	8 (27.6%)	<0.001	0.003
⩾ 1	38 (42.7%)	17 (28.3%)	21 (72.4%)		
Possibility of observation-only approach in cervical pregnancy					
Yes	6 (6.7%)	4 (6.7%)	2 (6.9%)	>0.9	>0.9
No	83 (93.3%)	56 (93.3%)	27 (93.1%)		
Preferred approaches for the management of diagnosed cervical pregnancy (multiple responses permitted)					
No experience/transfer the case to another department	26 (23.1%)	22 (36.7%)	4 (13.8%)	0.028	0.12
Systemic MTX i.m. administration	23 (25.8%)	11 (18.3%)	12 (41.4%)	0.037	0.1
Local MTX administration	15 (16.9%)	6 (10.0%)	9 (31.0%)	0.018	0.078
Misoprostol administration (vaginally, rectally)	15 (16.9%)	10 (16.7%)	5 (17.2%)	>0.9	>0.9
S&C	54 (60.7%)	32 (53.3%)	22 (75.9%)	0.063	0.2
Hysteroscopic resection	17 (19.1%)	10 (16.7%)	7 (24.1%)	0.4	0.6
Insertion of a balloon into the cervical canal for several days	1 (1.1%)	0 (0.0%)	1 (3.4%)	0.3	0.5
Laparoscopic surgery	1 (1.1%)	0 (0.0%)	1 (3.4%)	0.3	0.5
Combination of laparoscopic and vaginal approach	4 (4.5%)	3 (5.0%)	1 (3.4%)	>0.9	>0.9
Laparoscopic or laparotomic surgery	9 (10.1%)	6 (10.0%)	3 (10.3%)	>0.9	>0.9
Preferred approaches for the management of diagnosed cervical pregnancy (multiple responses permitted)					
No experience	26 (23.1%)	22 (36.7%)	4 (13.8%)	0.028	0.12
Pharmacological treatment	38 (42.7%)	21 (35.0%)	17 (58.6%)	0.042	0.082
Vaginal surgery	58 (65.2%)	34 (56.7%)	24 (82.8%)	0.018	0.056
Peritoneal surgery	13 (14.6%)	8 (13.3%)	5 (17.2%)	0.8	0.8
Hysterectomy performed for cervical pregnancy in the past 5 years					
Yes	2 (2.3%)	1 (1.7%)	1 (3.4%)	0.5	0.7
No	87 (97.7%)	59 (98.3%)	28 (96.6%)		
Clinical practice of uterine artery ligation for hemorrhage control in cases of large cervical pregnancy or CSP					
Yes	13 (14.6%)	5 (8.3%)	8 (27.6%)	0.024	0.087
No	76 (85.4%)	55 (91.7%)	21 (72.4%)		
PUL section					
Preferred approach to PUL with falling hCG levels					
Observation	74 (83.2%)	45 (75.0%)	29 (100.0%)	0.002	0.018
Active management	15 (16.8%)	15 (25.0%)	0 (0.0%)		
Preferred approach to PUL with not falling hCG levels					
Diagnostic laparoscopy ± RCUI	51 (57.3%)	37 (61.7%)	14 (48.3%)	0.3	0.5
Other management	38 (42.7%)	23 (38.3%)	15 (51.7%)		

hpy: hysterectomies per year; MTX: methotrexate; hCG: human chorionic gonadotropin; RCUI: revisio cavi uteri instrumentalis; S&C: suction and curettage; PUL: pregnancy of unknown location; i.m.: intramuscular injection.

Methotrexate was significantly more commonly available in larger centers (48.3% in Group S/M versus 82.8% in Group L/X; *p* = 0.002, *q* = 0.02, Table 3). However, the overall frequency of MTX use in the treatment of various types of ectopic pregnancy (EP) did not significantly differ by institutional size (Table 2). MTX was occasionally used for the treatment of any type of EP in 53.9% (48/89) of all institutions (45.0% in Group S/M versus 72.4% in Group L/X; *p* = 0.05, *q* = 0.13). Approximately three-quarters of physicians (71.9%) across all institution sizes recommended postponing pregnancy for at least 6 months following MTX administration.

In managing the most common EP localization—tubal pregnancy—38.2% (34/89) of institutions consistently employed surgical treatment, while 61.8% (55/89) reported occasional use of conservative strategies (observation or MTX). Salpingotomy was performed occasionally in 38.2% (34/89) of institutions, most frequently in the smallest centers (50.0% in Group S, Table 2); however, this difference was not statistically significant. In contrast, a statistically significant difference was observed regarding the use of dilation and curettage of the uterine cavity during surgery for tubal EP: 11.7% of smaller departments (Group S/M) reported never performing dilation and curettage, compared with 27.6% of larger institutions (Group L/X; *p* = 0.021, *q* = 0.085, Table 3). MTX was used occasionally in the treatment of tubal EP in 18.0% of institutions (16/89), but only one center reported using it in more than 20% of cases. The frequency of MTX use for tubal EP did not differ significantly between smaller and larger institutions (15.0% in Group S/M versus 24.1% in Group L/X; *p* = 0.4, *q* = 0.5, Table 3).

Laparoscopic surgery was the preferred treatment for interstitial pregnancy in 86.5% (77/89) of institutions across all sizes. Notably, larger institutions (Group L/X; large + extra large, >200 hpy) aimed to manage 100% of interstitial pregnancies laparoscopically, while 20% of smaller departments (Group S/M; small + medium, ⩽200 hpy) reported never choosing this approach (*p* = 0.007, *q* = 0.036, Table 3). Interestingly, 20.2% (18/89) of respondents reported occasionally preserving the fallopian tube during surgical treatment of interstitial pregnancy, significantly more often in larger centers (11.3% in Group S/M versus 41.4% in Group L/X; *p* = 0.004, *q* = 0.023, Table 3).

The survey revealed that 23.6% (21/89) of institutions had not treated a single case of CSP in the past 5 years, and 57.3% (51/89) had not encountered any cervical pregnancy (CP). Lack of clinical experience with CP was predominantly observed among smaller institutions. Only 26.7% of Group S/M institutions had treated three or more CSP cases in the previous 5 years, compared with 62.1% in Group L/X (*p* = 0.003, *q* = 0.021). Similarly, 28.3% of Group S/M institutions had treated at least one CP case, in contrast to 72.4% in Group L/X (*p* < 0.001, *q* = 0.003, Table 3). This disparity in clinical experience was also reflected in institutional willingness to treat these conditions: 41.7% of Group S/M institutions would refer a patient with CSP to another facility, compared with 10.3% in Group L/X (*p* = 0.003, *q* = 0.021), and 36.7% versus 13.8%, respectively, for CP (*p* = 0.028, *q* = 0.12, Table 3).

Across all institutions, the most commonly used treatment method for CSP was S&C in 47.2% of cases, and for CP in 60.7%. The use of S&C for CSP differed significantly by institutional size (36.7% in Group S/M versus 69.0% in Group L/X; *p* = 0.006, *q* = 0.033), while no statistically significant difference was observed for CP (53.3% in Group S/M versus 75.9% in Group L/X; *p* = 0.063, *q* = 0.02). For CSP, significant differences between institution sizes were observed in the use of vaginal surgery (43.3% in Group S/M versus 75.9% in Group L/X; *p* = 0.006, *q* = 0.020) and peritoneal approaches (laparoscopic or laparotomic; 25.0% in Group S/M versus 62.1% in Group L/X; *p* = 0.001, *q* = 0.006). Conversely, pharmacologic treatment of CSP did not differ significantly (26.7% in Group S/M versus 41.4% in Group L/X; *p* = 0.2, *q* = 0.3, Table 3). In the case of CP, statistical differences were modest: Pharmacologic treatment was reported by 35.0% of Group S/M and 58.6% of Group L/X (*p* = 0.042, *q* = 0.082); vaginal surgery was reported by 56.7% of Group S/M and 82.8% of Group L/X (*p* = 0.018, *q* = 0.056). Peritoneal surgery was the least common approach in both groups (13.3% in Group S/M versus 17.2% in Group L/X; *p* = 0.8, *q* = 0.8, Table 3). Notably, 14.6% (13/89) of institutions reported the occasional use of intraoperative uterine artery ligation for hemorrhage control during surgical treatment of CP (8.3% in Group S/M versus 27.6% in Group L/X; *p* = 0.024, *q* = 0.087, Table 3). Hysterectomy for CSP or CP was extremely rare, reported in only 3 (3.4%) and 2 (2.3%) cases, respectively, across the entire cohort. Experience with hysterectomy for CSP was limited to larger institutions (0.0% in Group S/M versus 10.3% in Group L/X; *p* = 0.032, *q* = 0.1, Table 3), while one case of hysterectomy for CP was reported in each group.

In the management of PUL, clinical approach varied based on the trend in hCG levels. When hCG values were declining, observation without active intervention was the preferred strategy in 83.2% (74/89) of institutions, including 100% of Group L/X and 75% of Group S/M (*p* = 0.003, *q* = 0.018, Table 3). When hCG levels were not declining, diagnostic laparoscopy was the most frequently reported approach across all institution sizes, used by 47.2% (42/89) of respondents (Table 2). Diagnostic laparoscopy with or without dilation and curettage was chosen by 61.7% of Group S/M (Small + Medium, ⩽200 hpy) and 48.3% of Group L/X (Large + Extra large, >200 hpy) institutions (*p* = 0.3, *q* = 0.5, Table 3).

## Discussion

Ectopic pregnancy (EP), with a frequency of 1%–2% of all pregnancies, is not an uncommon diagnosis.^1,4,6,8,10,15,17,57–60^ This condition is encountered by physicians across gynecological departments of all sizes and frequently presents as an emergency requiring prompt intervention. Our questionnaire-based study assessed approaches to the management of different types of EP across the Czech Republic, achieving a response rate of 96.7% from all registered gynecological departments. Responses to individual questions were statistically compared between institutions of different sizes based on the annual number of hysterectomies performed.

The therapeutic approach to EP primarily varies according to the experience of the treating institution, which often correlates with its size. Tubal pregnancy remains the most frequent and is generally considered the most straightforward type of EP to manage surgically. One-third of all departments (36.0%) manage this condition exclusively through surgery, while the remainder also occasionally employ observation or pharmacological treatment. Salpingectomy is consistently performed by three-fifths of surgeons (61.8%), whereas salpingotomy is occasionally employed by 38.2%. No statistically significant differences in these practices were observed based on the size of the institution.

Current practices vary globally. Some studies report a salpingotomy rate of 40%^57^ to 70%,^61,62^ whereas others refer exclusively to salpingectomy as the surgical method.^59,63–65^ A retrospective analysis of 62,588 women undergoing surgery for tubal EP in the United States between 2006 and 2015 showed a decrease in salpingotomy from 13.0% in 2006 to 6.0% in 2015 (*p* < 0.001).^45^ A population-based retrospective cohort study including 17,090 cases of tubal ectopic pregnancy in Ontario, Canada (2008–2019), reported that salpingotomy was performed in 10.5% of surgically treated patients.^66^ According to the Royal College of Obstetricians and Gynaecologists (RCOG), salpingectomy is preferred over salpingotomy (EBM grade B), with the latter considered for women with a damaged contralateral fallopian tube (EBM grade C).^58^ Salpingectomy may reduce the risk of repeat ectopic pregnancies in women undergoing In Vitro Fertilization (IVF) as well as in those who conceive spontaneously^62,67^ In summary, salpingotomy may improve IUP rates in younger women without a healthy contralateral tube. Therefore, treatment decisions should be strongly individualized.

Only 18% of all departments reported active use of systemic MTX for the treatment of tubal EP (Table 2). According to the current literature, MTX treatment successfully resolves EPs in approximately 70%–95% of cases without the need for surgery, with reduced success observed in women with higher initial hCG levels.^1,64^ When patients are properly selected, systemic MTX has comparable efficacy to laparoscopic salpingotomy in terms of tubal patency and future IUP rates.^17,62,68^ On the other hand, some studies report a statistically higher likelihood of conceiving by in vitro fertilization after salpingectomy than after MTX treatment (55.2% versus 18.0%, *p* < 0.001).^69^

Globally—and across many European countries—methotrexate is used substantially more frequently, although practices vary widely between nations. In several countries, including Turkey (63%),^65^ Oman (63%),^70^ Canada (51%),^66^ and Israel (51%),^69^ MTX is employed in more than half of tubal ectopic pregnancy cases. In contrast, a recent study from Ireland reported MTX use in only one-quarter (25%) of tubal ectopic pregnancies.^59^ Similarly, two Italian series documented MTX administration in 22%^41^ and 33% of cases.^64^ Practice patterns in the Czech Republic appear comparable to those in neighboring Poland, where MTX was used in 12% of women treated for tubal ectopic pregnancy.^61^ At the opposite end of the spectrum, a study from India reported MTX administration in just one of 75 patients with tubal ectopic pregnancy.^63^ In the United States, MTX use for tubal ectopic pregnancy increased from 14.5% in 2006 to 27.3% in 2015 (*p* < 0.001), with the highest uptake in high-volume and teaching hospitals.^45^ This pattern was only partially reflected in our data: 24% of large centers in Group L/X (Large + Extra large groups, >200 hpy) and 15% of smaller units in Group S/M (Small + Medium, ⩽200 hpy) reported intermittent MTX use, although this difference did not reach statistical significance (*p* = 0.4; *q* = 0.5; Table 3).

Observational management (i.e., conservative management without medical or surgical intervention) is also commonly used under appropriate conditions, with 64% of institutions of all sizes reporting occasional use of this approach (Table 3). Most published studies do not present observational management as a treatment option for tubal ectopic pregnancy.^61,63,64,69,70^ Others report only minimal use—approximately 7.5% in Dur et al.^65^—whereas some centers practice observational management successfully in 20%–30% of cases.^41,59^ According to RCOG, MTX treatment should only be offered after a definitive diagnosis has been made (EBM grade B), and observational management may be appropriate in women with initial hCG levels <1500 IU/L showing a declining trend (EBM grade B).^58^

Although laparoscopic surgery remains the overwhelmingly predominant treatment modality for interstitial pregnancy—used in 86.5% of reported cases—systemic or local MTX is employed only infrequently (Table 2). This contrasts markedly with practice in France, where 90% of stable interstitial ectopic pregnancies are managed medically with MTX (30% intramuscular only, 28% in situ only, 32% combined).^71^ A similar strategy was reported in Hong Kong, where surgery was reserved exclusively for unstable, ruptured, or MTX-failure cases.^6^ Globally, however, management practices vary substantially. In the United Arab Emirates and Australia, approximately half of interstitial pregnancies were managed surgically and half medically.^72,73^ In contrast, the study from Japan reported that all interstitial ectopic pregnancies were treated surgically.^57^

While larger centers (Group L/X) aim to manage all such cases laparoscopically, 20% of smaller institutions (Group S/M) report never performing laparoscopy for this condition (*p* = 0.007, *q* = 0.036, Table 3). Laparoscopic management of interstitial pregnancy is technically demanding and should be reserved for experienced surgeons due to the risk of significant intraoperative hemorrhage if the adjacent ascending branch of the uterine artery is damaged. Such expertise may not always be available in smaller healthcare facilities. Notably, laparoscopic surgery is associated with a shorter hospital stay (*p* < 0.0001), while no statistically significant differences have been observed in operative time (*p* = 0.50), blood loss (*p* = 0.93), surgical complications (*p* = 0.68), or the need for transfusion (*p* = 0.30) when compared with laparotomy.^74^ Interestingly, preservation of the fallopian tube during surgery for interstitial pregnancy is practiced by 20.2% of surgeons, with significantly higher rates in larger centers (11.3% in Group S/M versus 41.4% in Group L/X; *p* = 0.004, *q* = 0.023, Table 3). Surgical options include cornual (wedge) resection or cornuotomy, which is associated with reduced tissue disruption and potentially better reproductive outcomes.^4,75^

Nearly half (47.2%) of responding centers prefer ultrasound-guided S&C for CSP treatment. This approach is significantly more common in larger centers (69.0% in Group L/X versus 36.7% in Group S/M; *p* = 0.006, *q* = 0.033, Table 3), likely reflecting greater diagnostic experience. Other frequently utilized methods include hysteroscopic resection (25.8%) and systemic MTX administration (20.2%). This wide variability in therapeutic strategies is also reflected in the published literature, and a global consensus on optimal CSP management has not yet been established.^4,6,17,25,26^ At least 14 different treatment modalities have been described.^12^ In France, only 10% of clinicians reported using ultrasound-guided S&C for CSP, whereas 27% occasionally administered mifepristone.^71^

Standalone laparoscopic or open surgery is rarely used in the Czech Republic (15.7%), whereas the combined laparoscopic and vaginal approach is more frequent (23.6%), particularly in larger centers (37.9% in Group L/X versus 16.7% in Group S/M; *p* = 0.035, *q* = 0.1, Table 3). Laparoscopic resection of the uterine scar containing the ectopic pregnancy, followed by defect repair, has achieved a success rate of 97%.^12,27^ Across the available literature, roughly half of surgeons prefer vaginal S&C or hysteroscopic resection, whereas the other half employ laparoscopic or laparotomic surgery.^72,73,76,77^

Systemic MTX is occasionally used (20.2%), more frequently in larger facilities (Table 2). Nevertheless, MTX is described as a first-line therapy in most published cohorts.^6,72,73,76,77^ In France, according to the national survey, 80% of CSP cases are treated medically with MTX, divided approximately into one-third intramuscular only, one-third in situ, and one-third combined administration.^71^ However, the SMFM advises against systemic MTX as monotherapy due to complication rates as high as 54%.^37^

Only one center in Group L reported using a conservative technique involving double-balloon catheter placement at the site of the CSP, as described in the literature.^33^ Observational management of CSP is not practiced in the Czech Republic, and globally it is used only exceptionally,^73,76^ given the very high risk of severe complications, including uterine rupture with massive hemorrhage (up to 50%) and emergency hysterectomy in 25%–50% of ongoing pregnancies.^35^

Therapeutic approaches to CP largely mirror those for CSP. The majority of institutions use S&C (60.7%), followed by systemic (25.8%) and local (16.9%) MTX administration, as well as hysteroscopic resection (19.1%). Larger centers (Group L/X) employ MTX more frequently, both systemically (41.4%) and locally (31.0%; Table 3). A scoping review of 454 CP cases reported a success rate of 57.5% with systemic MTX therapy.^78^ S&C alone (OR 2.26), S&C combined with embolization (OR 4.85), and embolization alone (OR 5.17) demonstrated higher effectiveness. Notably, over half of patients (50.2%) required multiple interventions, and hysterectomy occurred in 9% of cases.^78^ This aligns with the broader literature, where most authors favor systemic, local, or combined MTX administration, in some cases supplemented with misoprostol.^71^ A retrospective analysis of 13 CP cases treated by local MTX under transvaginal ultrasound guidance reported a 92% success rate.^6^ However, according to the RCOG, the effectiveness of systemic MTX and surgical intervention for CP remains inconclusive (EBM grade D).^58^ Surgical intervention (laparoscopic or vaginal) and balloon tamponade are described only for selected cases.^72,73,77,79^ With increasing experience gained from CSP management, hysteroscopy may play an expanding role in the treatment of cervical ectopic pregnancies.^4^

Recent evidence demonstrates that the choice of treatment for ectopic pregnancy significantly influences both future fertility and the risk of recurrent ectopic pregnancy. Meta-analyses indicate that tubal-preserving surgery—particularly salpingotomy—is associated with higher IUP rates compared with salpingectomy, although it carries a moderately increased risk of recurrent ectopic pregnancy that necessitates careful follow-up.^44^ Data from ART populations similarly show that a history of EP increases the likelihood of IVF-related ectopic implantation, while bilateral salpingectomy reduces tubal EP but proportionally increases non-tubal cases.^67^ Population-level declines in EP incidence reported in Japan underscore the importance of modifiable factors such as smoking prevalence and chlamydial infection rates.^57^

Management of rare EP forms also varies internationally. High-volume centers report favorable outcomes with targeted medical therapies—including intralesional MTX—when supported by advanced imaging and specialized surgical expertise.^6^ Compared with surveys from the United Kingdom, Australia, France, Japan, and Hong Kong, Czech practice generally aligns in the selective use of medical or surgical treatment for non-tubal EPs.^71–73,76,77,79^ However, unlike these countries, the Czech Republic lacks national clinical guidelines, contributing to wider variability in MTX use, diagnostic approaches, and follow-up protocols. In addition, in the absence of formal referral pathways, the management of rare EP types is inconsistent, with some survey responses reflecting theoretical rather than routine practice. Harmonizing national care with international recommendations and establishing structured referral networks could improve consistency and support better reproductive outcomes.

Because CSP and CP are rare clinical entities, findings related to their management must be interpreted cautiously. More than half of surveyed institutions had not encountered a CP within the previous 5 years, and nearly one-quarter had not treated any CSP case. Consequently, practical experience—particularly in smaller centers—is limited, and reported strategies may reflect preferred rather than routinely applied approaches. This is a common limitation in questionnaire-based studies of rare conditions and highlights the need for centralized expertise and clear referral pathways.

Population-based differences further contribute to the institutional variability observed. Large tertiary centers in metropolitan regions manage a higher volume and broader spectrum of ectopic pregnancies, including rare non-tubal cases, and therefore report wider access to medical therapies and minimally invasive interventions. Smaller regional hospitals encounter fewer complex presentations and more often rely on surgical management or interhospital transfer. These large–small institutions′ differences reinforce the need for national guidelines to ensure consistent care regardless of institutional size.

In the Czech Republic, the absence of unified national guidelines has led to substantial variability in diagnostic and therapeutic management. Although differences in the use of MTX, surgical techniques, and approaches to both tubal and non-tubal EPs have been recognized, they have not previously been assessed systematically at the national level. To address this gap, we conducted a nationwide questionnaire-based survey evaluating management strategies for all major EP types, stratified by institutional size. This study therefore provides the first comprehensive national overview of ectopic pregnancy management across Czech inpatient gynecological departments.

The considerable variability in ectopic pregnancy care across Czech institutions underlines the need for national standardization. Developing evidence-based guidelines aligned with ESHRE and RCOG recommendations, establishing referral centers for rare or complex EPs, and expanding specialized training in early pregnancy ultrasound and methotrexate protocols could support smaller hospitals and promote consistent, high-quality management nationwide.^7,58,60^

### Limitations

The primary limitation of this study is its restriction to the Czech Republic, a country with approximately 11 million inhabitants and an estimated 1500 ectopic pregnancies per year. Another limitation arises from the rarity of certain non-tubal ectopic pregnancy types, meaning that many clinicians may have limited personal experience with these entities. As a result, some responses likely reflect theoretical knowledge rather than direct clinical practice. The absence of formal psychometric validation of the questionnaire may also be considered a limitation of the study; however, the instrument was pilot-tested in a prior nationwide study involving approximately half of the target population and was subsequently refined based on this experience.^53^ Finally, no a priori sample size calculation or power analysis was performed, as the study was designed as a nationwide census survey of all eligible institutions rather than as a sample-based study; however, subgroup analyses—particularly for rare ectopic pregnancy types—may still have been underpowered.

Nonetheless, the study has a major strength: an exceptionally high response rate, with data obtained from 96.7% of all eligible healthcare facilities, regardless of size. This far-exceeds the response rates of comparable surveys.^71^ Consequently, the results provide a robust and comprehensive overview of current management patterns for ectopic pregnancy throughout the Czech Republic. These findings may also be informative for neighboring Central European countries with comparable healthcare systems and may support the refinement of practical clinical protocols for the management of both tubal and non-tubal ectopic pregnancies.

## Conclusion

In the management of ectopic pregnancies, 46% of healthcare institutions in the Czech Republic have never utilized methotrexate. Furthermore, only 18% of centers reported the use of systemic MTX for the treatment of intact tubal ectopic pregnancies. Surgery remains the primary therapeutic approach for tubal ectopic pregnancy, with salpingotomy performed by 38.2% of surgeons at least occasionally. Laparoscopic surgery was the preferred modality for interstitial pregnancies, employed in 86.5% of cases, with full implementation observed in large tertiary centers. For cesarean scar and cervical ectopic pregnancies, S&C under ultrasound guidance was the most commonly used approach, although a wide variety of management strategies were reported. This nationwide survey revealed substantial heterogeneity in the management of ectopic pregnancies across gynecological departments within one country with a homogeneous healthcare system, largely influenced by institutional size and available resources. The findings highlight the need for the development of standardized national guidelines to ensure consistent and evidence-based care for ectopic pregnancies.

## Supplemental Material

sj-doc-1-whe-10.1177_17455057261435815 – Supplemental material for Ectopic pregnancy management and treatment strategies: A nationwide survey of Czech gynecological facilities Supplemental material, sj-doc-1-whe-10.1177_17455057261435815 for Ectopic pregnancy management and treatment strategies: A nationwide survey of Czech gynecological facilities by Borek Sehnal, Jan Zapletal, Michael J. Halaska, Krystof Brecka, Barbora Svedova, Martin Hruda, Anna Babkova, Lukas Rob and Petr Waldauf in Women's Health

sj-docx-2-whe-10.1177_17455057261435815 – Supplemental material for Ectopic pregnancy management and treatment strategies: A nationwide survey of Czech gynecological facilities Supplemental material, sj-docx-2-whe-10.1177_17455057261435815 for Ectopic pregnancy management and treatment strategies: A nationwide survey of Czech gynecological facilities by Borek Sehnal, Jan Zapletal, Michael J. Halaska, Krystof Brecka, Barbora Svedova, Martin Hruda, Anna Babkova, Lukas Rob and Petr Waldauf in Women's Health

sj-docx-3-whe-10.1177_17455057261435815 – Supplemental material for Ectopic pregnancy management and treatment strategies: A nationwide survey of Czech gynecological facilities Supplemental material, sj-docx-3-whe-10.1177_17455057261435815 for Ectopic pregnancy management and treatment strategies: A nationwide survey of Czech gynecological facilities by Borek Sehnal, Jan Zapletal, Michael J. Halaska, Krystof Brecka, Barbora Svedova, Martin Hruda, Anna Babkova, Lukas Rob and Petr Waldauf in Women's Health
