# Supplementary material for: Ectopic pregnancy management and treatment strategies: A nationwide survey of Czech gynecological facilities
Source: Womens Health (Lond). 2026 Apr 18;22:17455057261435815. doi: 10.1177/17455057261435815 (PMC13100430; doi:10.1177/17455057261435815)
Supplement: sj-doc-1-whe-10.1177_17455057261435815 – Supplemental material for Ectopic pregnancy management and treatment strategies: A nationwide survey of Czech gynecological facilities [file sj-doc-1-whe-10.1177_17455057261435815.doc]

Table S1. STROBE Statement—Checklist

|  | Item No | Recommendation | Reported on page No |
| --- | --- | --- | --- |
| **Title and abstract** | 1 | (*a*) A nationwide cross-sectional observational questionnaire study | 1 |
|  |  | (*b*) Abstract (Bacround, Objectives, Design, Methods, Results, Conclusion) | 3 |
| Introduction | | |  |
| Background/rationale | 2 | Introduction, paragraphs 1–4 (clinical relevance, epidemiology, heterogeneity of EP management, lack of national data) | 4-11 |
| Objectives | 3 | End of Introduction / Aim of the Survey section: Explicit statement of objectives; no prespecified hypotheses (descriptive survey) | 11 |
| Methods | | |  |
| Study design | 4 | Methods, first paragraph: Nationwide cross-sectional, questionnaire-based observational study | 11 |
| Setting | 5 | Methods: All inpatient gynecological departments in the Czech Republic, Data collection January–March 2025 | 11-12 |
| Participants | 6 | Methods – Inclusion and exclusion criteria: All 92 inpatient gynecological departments providing 24/7 acute care, Institutional-level participation | 13 |
| Variables | 7 | Methods – Questionnaire description: Diagnostic approaches, Treatment strategies for tubal, interstitial, CSP, CP, and PUL, Institutional characteristics (hysterectomy volume) | 11-12 |
| Data sources/ measurement | 8 | Methods – Questionnaire development and Delphi process: Expert consensus, Standardized terminology (ESHRE definitions), Google Forms platform | 11-12 |
| Bias | 9 | Discussion – Study limitations: Potential reporting bias, Limited experience with rare EP types, Institutional-level responses may reflect preferred rather than actual practice | 22 |
| Study size | 10 | Methods – Sample size and study population: Nationwide census survey, All eligible institutions invited, No formal sample size calculation performed | 12-13 |
| Quantitative variables | 11 | Statistical analysis: Categorization by hysterectomy volume (S/M/L/X), Justification provided | 13-14 |
| Statistical methods | 12 | (*a*) Statistical analysis: Fisher’s exact test with Monte Carlo simulation, False Discovery Rate (FDR) correction | 13 |
|  |  | (*b*) Statistical analysis: Comparisons by institutional size (S/M vs L/X) | 13 |
|  |  | (*c*) Statistical analysis: Pairwise deletion, No imputation applied | 13 |
|  |  | (*d*) Not applicable, Census survey (not sample-based) |  |
|  |  | (*e*) Not applicable, Descriptive institutional survey |  |
| Results | | |  |
| Participants | 13 | (a) Results – first paragraph: 92 eligible institutions, 89 responded (96.7%) | 14 |
|  |  | (b) Methods – recruitment process: Non-responders contacted by phone, Non-responders (n=3) identified; no reasons provided due to anonymity. | 12 |
|  |  | (c) Flow diagram is attached as Figure 1. Study design: stepwise modified Delphi method used to achieve consensus on the questionnaire regarding ectopic pregnancy management and treatment strategies. | 11-12  Figure 1 |
| Descriptive data | 14 | (a) Results – Tables 1–3: Institutional characteristics, Volume categories, Treatment availability | 14-16  Tables 1–3 |
|  |  | (b) Statistical analysis: Missing data handled by pairwise deletion | 13 |
| Outcome data | 15 | Results: Frequencies and percentages of management strategies |  |
| Main results | 16 | (*a*) Results – Tables 2 and 3: Group comparisons, p-values and q-values reported; no confidence intervals required for proportions. | 14-16  Tables 2-3 |
|  |  | (*b*) Methods – Institutional volume classification: Hysterectomy volume groups defined (≤100, 101–200, 201–300, >300). | 13 |
|  |  | (*c*) Not applicable (No patient-level risk estimation) |  |
| Other analyses | 17 | Results: Subgroup analyses by institutional size. No interactions or sensitivity analyses applicable. |  |
| Discussion | | |  |
| Key results | 18 | Discussion: Results summarized in relation to objectives,  Substantial heterogeneity in the management of ectopic pregnancies across gynecological department, largely influenced by institutional size | 16-21 |
| Limitations | 19 | Limitations section: National scope-restriction to the Czech Republic. Limited personal experience with rare non-tubal ectopic pregnancy types, No power calculation, Institutional-level data (theoretical knowledge) | 22-23 |
| Interpretation | 20 | Discussion – full section: Comparison with international literature, Clinical implications | 16-22 |
| Generalisability | 21 | Discussion – late paragraphs: Applicability to similar healthcare systems, Central European context | 21-22 |
| Other information | | |  |
| Funding | 22 | Funding section: Cooperatio program, Maternal and Childhood Care No. 207035 (Third Faculty of Medicine, Charles University, Prague), Grant Nr. NW24-09-00505 (Ministry of Health of the Czech Republic). No role of funders in study design or analysis . No salaries were paid. | 25 |

**Note:** An Explanation and Elaboration article discusses each checklist item and gives methodological background and published examples of transparent reporting. The STROBE checklist is best used in conjunction with this article (freely available on the Web sites of PLoS Medicine at http://www.plosmedicine.org/, Annals of Internal Medicine at http://www.annals.org/, and Epidemiology at http://www.epidem.com/). Information on the STROBE Initiative is available at www.strobe-statement.org.
