# Supplementary material for: Ectopic pregnancy management and treatment strategies: A nationwide survey of Czech gynecological facilities
Source: Womens Health (Lond). 2026 Apr 18;22:17455057261435815. doi: 10.1177/17455057261435815 (PMC13100430; doi:10.1177/17455057261435815)
Supplement: sj-docx-2-whe-10.1177_17455057261435815 – Supplemental material for Ectopic pregnancy management and treatment strategies: A nationwide survey of Czech gynecological facilities [file sj-docx-2-whe-10.1177_17455057261435815.docx]

Figure S1. Trends in spontaneous and elective (induced) abortions and ectopic pregnancies in the Czech Republic (1990–2021)


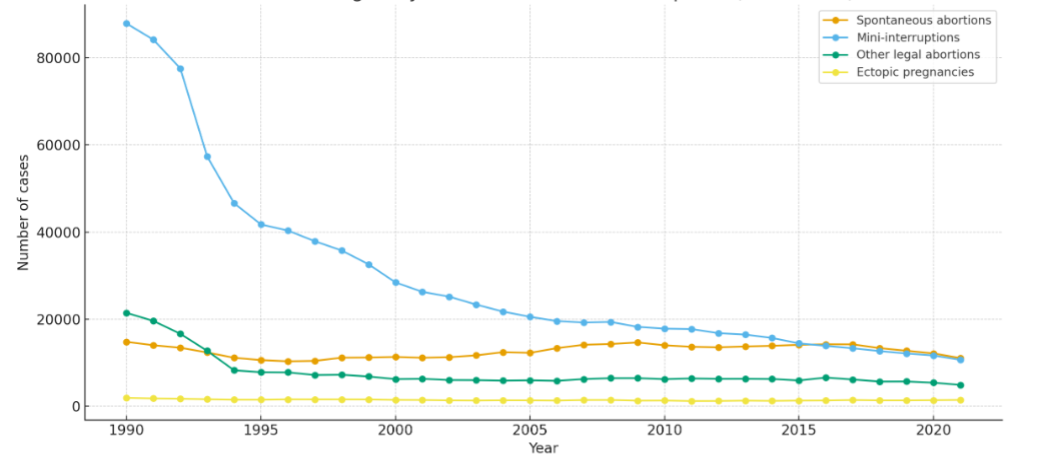


Term definitions:

Spontaneous abortion: natural loss of a pregnancy before 24 weeks of gestation.

Mini-interruptions: elective (induced) abortions performed on request up to 8 weeks of gestation.

Other legal abortions: elective (induced) abortions performed up to 24 weeks of gestation (on the woman’s request up to 12 weeks, or for medical indications with the woman’s consent up to 24 weeks).

Ectopic pregnancies: implantation of a fertilized ovum outside the uterine cavity.
