# Supplementary material for: Ectopic pregnancy management and treatment strategies: A nationwide survey of Czech gynecological facilities
Source: Womens Health (Lond). 2026 Apr 18;22:17455057261435815. doi: 10.1177/17455057261435815 (PMC13100430; doi:10.1177/17455057261435815)
Supplement: sj-docx-3-whe-10.1177_17455057261435815 – Supplemental material for Ectopic pregnancy management and treatment strategies: A nationwide survey of Czech gynecological facilities [file sj-docx-3-whe-10.1177_17455057261435815.docx]

Table S2. Questions and response options from the online questionnaire

| Question | Response |
| --- | --- |
| General information Section | |
| Name of the inpatient medical facility providing 24-hour acute gynecological care | Optional |
|  |  |
| Number of residents in the municipality where the medical facility is located | <10 000 |
|  | 10 000 – 20 000 |
|  | 20 000 – 30 000 |
|  | 30 000 – 50 000 |
|  | 50 000 – 100 000 |
|  | 100 000 – 1 000 000 |
|  | > 1 000 000 |
|  | |
| State accreditation of medical facility | Wihout accreditation |
|  | Akreditace type I |
|  | Akreditace type II |
|  | Akreditace type III |
|  | |
| Number of births in 2023 | No maternity hospital |
|  | ≤600 |
|  | 601 - 1000 |
|  | 1001 - 2000 |
|  | 2 001 – 3 000 |
|  | >3000 |
|  | |
| Number of births in 2024 | No maternity hospital |
|  | ≤600 |
|  | 601 - 1000 |
|  | 1001 - 2000 |
|  | 2 001 – 3 000 |
|  | >3000 |
|  | |
| Number of hysterectomies in 2024 | ≤100 |
|  | 101 - 200 |
|  | 201 - 300 |
|  | >300 |
|  | |
| Availability of chemotherapy in a medical facility | No |
|  | Yes |
|  | |
| Availability of Methotrexate in a medical facility | No |
|  | Yes |
|  | |
| Intact Tubal Pregnancy Section | |
| General approach in case of intact tubal pregnancy | Always surgery |
|  | Sometimes pharmacological treatment |
|  | |
| Type of surgical procedure for intact small tubal ectopic pregnancy | Always salpingectomy |
|  | Sometimes salpingostomy |
|  | |
| Criteria for observational management in case of small tubal pregnancy  Multiple responses permitted | Always active management (surgery/methotrexate) |
|  | hCG level only |
|  | Ultrasound signs (size, free fluid etc.) |
|  | |
| Criteria for methotrexate treatment in case of tubal pregnancy  Multiple responses permitted | Never |
|  | hCG level |
|  | Ultrasound signs (size, free fluid etc.) |
|  | |
| How often do you use methotrexate to treat tubal pregnancy | Never |
|  | ≤5 % |
|  | 5 - 20 % |
|  | >20 % |
|  | |
| Performing revision curettage of the uterine cavity (RCUI), , also referred to as dilation and curettage (D&C), in the context of tubal pregnancy management | Never |
|  | Sometimes (in case of bleeding, etc.) |
|  | Always |
|  | |
| Use of methotrexate in the treatment of some types of ectopic pregnancy  Multiple responses permitted | Never |
|  | Systemic application (i.m.) in tubal pregnancy |
|  | Systemic application (i.m.) for atypical localizations (CSP, interstitial, PUL, etc.) |
|  | Local application (with a needle into the embryo) in atypical locations (e.g. CSP) |
|  | |
| How long do you recommend a patient wait to get pregnant after using last methotrexate dose? | Anytime |
|  | in 3 months |
|  | in 6 months or later |
|  | |
| Cervical Pregnancy (not cesarean scar pregnancy) Section | |
| Preferred type of surgical procedure for dg. Cervical pregnancy (not cesarean scar pregnancy)  Multiple responses permitted | Suction and Curettage (S&C) |
|  | Hysteroscopic resection |
|  | Insertion of a balloon into the cervical canal for several days |
|  | Laparoscopic surgery |
|  | Laparotomic surgery |
|  | Laparoscopic or laparotomic surgery |
|  | Systemic application (i.m.) of MTX |
|  | Local application of MTX |
|  | Misoprostol administration (vaginally, rectally) |
|  | Expectation / Observation |
|  | Transfer the case to another department |
|  | |
| Have you ever performed only observation without any other intervention for cervical pregnancy (not cesarean scar pregnancy)? | Yes |
|  | No |
|  | |
| Approximately how many cases of cervical pregnancy have you diagnosed during the last 5 years? | Number |
|  |  |
| Have you performed a hysterectomy for cervical pregnancy during the last 5 years? | Yes |
|  | No |
|  | |
| Cesarean Scar Pregnancy Section | |
| Preferred type of surgical procedure for dg. cesarean scar pregnancy  Multiple responses permitted | Suction and Curettage (S&C) |
|  | Hysteroscopic resection |
|  | Insertion of a balloon into the cervical canal for several days |
|  | Laparoscopic surgery |
|  | Laparotomic surgery |
|  | Laparoscopic or laparotomic surgery |
|  | Systemic application (i.m.) of MTX |
|  | Local application of MTX |
|  | Misoprostol administration (vaginally, rectally) |
|  | Transfer the case to another department |
|  | |
| Have you ever performed only observation without any other intervention for cesarean scar pregnancy? | Yes |
|  | No |
|  | |
| Approximately how many cases of cesarean scar pregnancy have you diagnosed during the last 5 years? | Number |
|  | |
| Have you performed a hysterectomy for cesarean scar pregnancy during the last 5 years? | Yes |
|  | No |
|  | |
| Do you ever use surgical uterine arteries ligation for large cervical gravidity or large cesarean scar pregnancy? | Yes |
|  | No |
|  | |
| Interstitial Pregnancy Section | |
| Preferred type of surgical procedure for dg. interstitial pregnancy (multiple responses permitted) | Laparoscopic surgery |
|  | Laparotomic surgery |
|  | Systemic application (i.m.) of MTX |
|  | Local application of MTX |
|  | Transfer the case to another department |
|  | |
| Performing salpingectomy in case of interstitial pregnancy | Always salpingectomy |
|  | Sometimes not |
|  | Transfer the case to another department |
|  | |
| PUL (Pregnancy of Unknown Location) Section | |
| Preferred approach in case of PUL (pregnancy of unknown location) if hCG levels are dropping | Observation |
|  | Diagnostic laparoscopy |
|  | Systemic application (i.m.) of MTX |
|  | Transfer the case to another department |
|  | |
| Preferred approach in case of PUL (pregnancy of unknown location) if hCG levels are not dropping | Observation |
|  | Diagnostic laparoscopy |
|  | Systemic application (i.m.) of MTX |
|  | Transfer the case to another department |

MTX - methotrexate; hCG - human chorionic gonadotropin; RCUI - revisio cavi uteri instrumentalis
